# Ocular blood flow decreases during passive heat stress in resting humans

**DOI:** 10.1186/1880-6805-32-23

**Published:** 2013-12-06

**Authors:** Tsukasa Ikemura, Akane Miyaji, Hideaki Kashima, Yuji Yamaguchi, Naoyuki Hayashi

**Affiliations:** 1 Graduate School of Human-Environment Studies, Kyushu University, Kasuga-koen 6-1, Kasuga, Fukuoka 816-8580, Japan; 2 Graduate School of Decision Science and Technology, Tokyo Institute of Technology, Ookayama 2-12-1-W9-1, Meguro, Tokyo 152-8552, Japan

**Keywords:** Retinal circulation, Choroidal circulation, Hyperthermia

## Abstract

**Background:**

Heat stress induces various physiological changes and so could influence ocular circulation. This study examined the effect of heat stress on ocular blood flow.

**Findings:**

Ocular blood flow, end-tidal carbon dioxide (*P*_ET_CO_2_) and blood pressure were measured for 12 healthy subjects wearing water-perfused tube-lined suits under two conditions of water circulation: (1) at 35°C (normothermia) for 30 min and (2) at 50°C for 90 min (passive heat stress). The blood-flow velocities in the superior temporal retinal arteriole (STRA), superior nasal retinal arteriole (SNRA), and the retinal and choroidal vessels (RCV) were measured using laser-speckle flowgraphy. Blood flow in the STRA and SNRA was calculated from the integral of a cross-sectional map of blood velocity. *P*_ET_CO_2_ was clamped at the normothermia level by adding 5% CO_2_ to the inspired gas. Passive heat stress had no effect on the subjects’ blood pressures. The blood-flow velocity in the RCV was significantly lower after 30, 60 and 90 min of passive heat stress than the normothermic level, with a peak decrease of 18 ± 3% (mean ± SE) at 90 min. Blood flow in the STRA and SNRA decreased significantly after 90 min of passive heat stress conditions, with peak decreases of 14 ± 3% and 14 ± 4%, respectively.

**Conclusion:**

The findings of this study suggest that passive heat stress decreases ocular blood flow irrespective of the blood pressure or arterial partial pressure of CO_2_.

## Background

The ocular circulation consists of the choroidal and retinal vasculatures. Both blood vessels branch from the ophthalmic artery [1]. The ocular circulation nourishes the retina, which plays an important role in vision [2]. We have previously reported that changes in retinal and choroidal blood flow are associated with a change in visual acuity [3]. In addition, concomitant increases in choroidal blood flow and contrast sensitivity were found in healthy subjects after sildenafil administration [4]. Thus, changes in ocular blood flow can influence the visual function.

Heat stress elevates body temperature, which can lead to hyperventilation and a consequent reduction in the arterial partial pressure of carbon dioxide (*P*aCO_2_) [5]. Since the retinal and choroidal blood vessels are sensitive to variations in *P*aCO_2_[2], heat stress may cause a reduction in flow in both ocular vessels. We previously reported that ocular blood flow in both vessels decreased concomitantly with hypocapnia during hyperventilation [3]; however, the effect of passive heat stress alone on ocular blood flow has yet to be examined.

It has been found that retinal blood flow decreased and choroidal blood flow was suppressed during exhaustive exercise both under hot and thermoneutral conditions [6]. This response was larger under the hot condition than in the thermoneutral condition, despite both conditions inducing a similar decrease in *P*aCO_2_, implying that additional passive heat stress can induce a further reduction in ocular blood flow. Thus, the purpose of the present study was to test the hypothesis that retinal and choroidal blood flow is decreased by passive heat stress for constant *P*aCO_2_. The flow response to passive heat stress in both of these ocular blood vessels was assessed under eucapnic conditions.

## Methods

### Subjects

The subjects were 12 healthy males (age, 24 ± 2 years (mean ± SD); height, 172 ± 8 cm; body mass 61 ± 6 kg). All of the subjects were free of any known autonomic dysfunction or cardiovascular or ocular disease, and were not taking any medication. The Ethics Committee of the Institution of Health Science, Kyushu University, Japan, approved the experimental protocol, and all subjects provided written informed consent to participate prior to the commencement of the study. All of the protocols used conformed to the Declaration of Helsinki.

### Protocol

The subjects were asked to abstain from caffeinated beverages and strenuous exercise for 6 h, and from eating for at least 2 h before the experiment. Following arrival at the laboratory, each subject put on a water-perfused tube-lined suit that covered the entire body except for the head, hands and feet. Initially, thermoneutral water (35°C) was circulated through the tube-lined suit for 30 min (normothermia). The subjects were then exposed to passive whole-body heat by raising the temperature of the circulating water to 50°C for 90 min. All subjects were required to maintain a resting state in a seated position throughout the experimental procedure.

Each subject’s blood pressure, finger skin blood flow (fSBF) and heart rate (HR) were recorded continuously throughout the trial. The respiratory variables, ocular blood velocity and external ear temperature were recorded every 10 min during normothermia, and at 30, 60 and 90 min after the induction of passive heat stress. In addition, to ensure that the end-tidal carbon dioxide (*P*_ET_CO_2_) remained stable, the respiratory variables were measured at 20, 50 and 80 min after the induction of passive heat stress. When hypocapnia was observed, 5% CO_2_ gas was added to the inspired gas until the *P*_ET_CO_2_ returned to the normothermic level. The subjects were asked to open their eyes without blinking for 6 s during the recording of the image for the ocular blood flow measurement.

### Measurements

The beat-by-beat blood pressure was monitored with an automatic sphygmomanometer attached to the left middle finger (Finometer, Finapres Medical Systems, Amsterdam, The Netherlands). An electrocardiogram was recorded continuously using a bioelectrical amplifier (MEG2100, Nihon-Kohden, Tokyo, Japan). The fSBF was measured using a laser Doppler flowmeter (FLO-CI, Omegawave, Tokyo, Japan). The temperature of the right external ear was measured using an infrared thermometer (Omron, Kyoto, Japan). The HR and mean arterial pressure (MAP) were calculated from the bioelectrical amplifier and blood-pressure recordings. The averaged data of the last minute in each of the measurement periods was used for analysis.

The subjects wore a nose clip and breathed through a mouthpiece that was connected to a hot-wire flowmeter (RM-300, Minato Medical Sciences, Osaka, Japan) to measure tidal volume (*V*_T_) and *P*_ET_CO_2_. The flowmeter was calibrated using a 2-l syringe. Samples of respired gas (1 ml/s) were regularly drawn from the mouthpiece and analyzed for CO_2_ using a mass spectrometer (WSMR-1400, Westron, Chiba, Japan), which was calibrated with fresh air and precision gases. *P*aCO_2_ was estimated from *V*_T_ and *P*_ET_CO_2_[7].

Laser-speckle images were obtained using a laser-speckle flowgraphy (LSFG) system (SoftCare, Fukuoka, Japan), which is described elsewhere (for example, [8]). The LSFG measurements were made in triplicate at each time point and the mean of the three values was used for analysis. Ocular blood-velocity data were obtained from the retinal and choroidal vasculature (RCV), the superior temporal retinal arteriole (STRA) and the superior nasal retinal arteriole (SNRA). The RCV accounts for most of the choroidal circulation, since the choroidal blood flow constitutes 85% of the total ocular blood flow to the retina [9]. The STRA and SNRA supply blood to the retina [1]. Blood flow in the STRA and SNRA was calculated from the integral of a cross-sectional map of blood velocity within the selected arteriole. The conductance index (CI) of each ocular vessel was calculated by dividing the ocular blood flow by the MAP.

### Data analysis

Data were expressed as mean ± SE values. The effects of time and blood vessels were examined by repeated-measures ANOVA. When a significant *F* value was detected, this was analyzed further against the baseline value using Scheffé’s *post hoc* test. The level of statistical significance was set at *P* < 0.05. All of the statistical analyses were performed using SAS (version 8.2, SAS Institute, Cary, NC, USA) at the Computing and Communications Center, Kyushu University, Japan.

## Results

During passive heat stress, HR, fSBF and external ear temperature increased significantly from the normothermia levels, while MAP and *P*aCO_2_ remained unchanged (Table 1).

**Table 1:** Parameters during normothermia and passive heat stress

**Parameter**	**Normothermia**^**a**^	**Passive heat stress**^**a**^		
		**30 min**	**60 min**	**90 min**
Heart rate (bpm)	69.14 ± 2.29	76.56 ± 3.01*	83.27 ± 3.61*	89.39 ± 4.36*
Finger skin blood flow (%)	—	49.53 ± 8.83*	66.30 ± 12.98*	52.39 ± 11.16*
Arterial partial pressure of CO_2_ (mmHg)	36.40 ± 0.74	35.73 ± 0.91	35.07 ± 0.97	35.55 ± 0.83
Mean arterial pressure *(*mmHg)	78.06 ± 2.52	76.40 ± 2.88	75.52 ± 2.61	77.07 ± 3.02
External ear temperature (°C)	36.09 ± 0.19	36.12 ± 0.19	36.34 ± 0.19*	36.48 ± 0.19*

^**a**^Values are means ± SE.

*P < 0.05 vs. normothermia.

**Figure 1:**
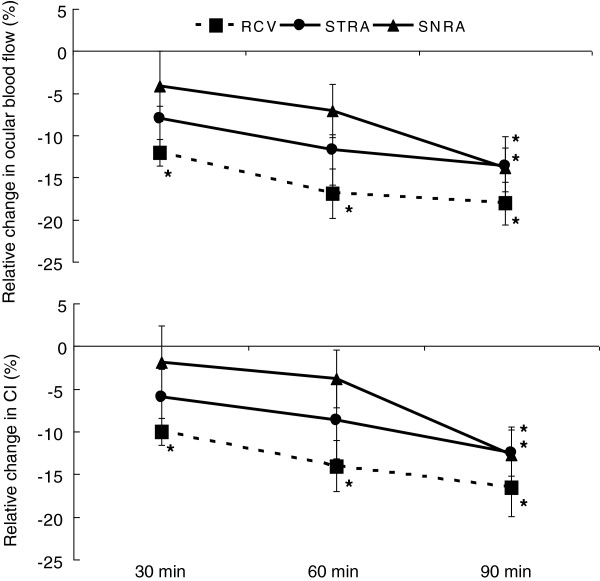
**Relative changes in ocular blood flow (upper panel) and conductance index (lower panel) during passive heat stress.** Values are means ± SE. CI, conductance index; RCV, retinal and choroidal vasculature; STRA, superior temporal retinal artery; SNRA, superior nasal retinal artery. *P < 0.05 vs. normothermia.

STRA and SNRA blood flow and RCV blood flow velocity decreased significantly from the normothermia level after 90 min of passive heat stress in all subjects (Figure 1). Only the RCV blood velocity decreased significantly throughout the heat stress condition. While the blood velocity in the RCV tend to be lower than that in the SNRA at 60 min of passive heat stress (*P* = 0.06), a significant difference was not observed among ocular vessels through the passive heat stress trial. The CI responses for all of the ocular vessels to the heat stress were similar to those of ocular blood flow (Figure 1).

## Discussion

The main finding of the present study was that passive heat stress reduced the blood flow and CI in the retinal and choroidal vessels, even though the *P*aCO_2_ remained unchanged. These results indicate that factors other than *P*aCO_2_ influence the ocular circulation in both the SNRA and STRA during passive heat stress.

Decreased ocular blood flow during passive heat stress can be attributed to a sympathetic nervous response. The cerebral blood flow, which is also sensitive to CO_2_, did not return to the baseline value on restoration of *P*_ET_CO_2_ in heat-stress conditions, suggesting that other factors, such as sympathetic nerve activity, may be sustaining the reduction in cerebral blood flow [10]. Sympathetic nerve activity is enhanced by heat stress [11]. The sympathetic nerve solely innervates the choroidal vessels in the ocular circulation. In turn, the ophthalmic artery, which is upstream of the choroidal and retinal vessels, has a rich sympathetic innervation [2,12]. Enhanced sympathetic activity may induce vasoconstriction in the ophthalmic artery, consequently decreasing the conductance of both ocular vessels during passive heat stress. This possibility is partly supported by the observed decrease in fSBF, which should be due to sympathetic activation.

Another factor that can cause a decrease in ocular blood flow is an alteration in the distribution of cardiac output $\left(\dot{Q}\right)$. $\dot{Q}$ dynamics influence the cerebral blood flow, independently of *P*aCO_2_ or cerebral perfusion pressure [13]. The ocular blood flow may also be affected by changes in $\dot{Q}$. More blood is distributed to the skin to aid heat dissipation during hyperthermia [14], which can result in a reduction in the distribution of blood to the ocular circulation during heat stress.

The choroidal circulation responds earlier to heat stress than the retinal circulation. This could be explained by autonomic innervation: as mentioned above, the autonomic innervation is richer for the choroidal circulation than for the retinal circulation [2]. Thus, it is likely that the sympathetic activation induced by heat stress influences the autonomic-rich choroidal circulation earlier than the retinal circulation.

In conclusion, the present results suggest that the blood flow in the retinal and choroidal vasculature is decreased by passive heat stress even without a change in *P*aCO_2_. These responses can be attributable to a heat-stress-induced enhancement of the sympathetic nerve activity and/or a redistribution of blood. The finding that the choroidal circulation was more responsive than the retinal circulation to heat stress could be explained by differences in sympathetic innervation between these two parts of the ocular circulation.

## Abbreviations

CI: Conductance index; fSBF: Finger skin blood flow; HR: Heart rate; LSFG: Laser-speckle flowgraphy; MAP: Mean arterial pressure; PaCO2: Arterial partial pressure of carbon dioxide; PETCO2: End-tidal carbon dioxide; $\dot{Q}$: Cardiac output; RCV: Retinal and choroidal vasculature; SNRA: Superior nasal retinal artery; STRA: Superior temporal retinal artery; VT: Tidal volume

## Competing interests

The authors declare that they have no competing interests.

## Authors’ contributions

AM, HK and YY contributed to the data collection and data interpretation. TI and NH contributed equally to the study design, data collection, data analysis, data interpretation and writing of the manuscript. All authors read and approved the final manuscript.
